# False-Positive *Clostridium difficile* in Negative-Control Reactions Peak and Then Decrease with Repetitive Refrigeration of Immunoassay

**DOI:** 10.1155/2014/128120

**Published:** 2014-09-11

**Authors:** Alexander Rodriguez-Palacios, Henry R. Stämpfli, Yung-Fu Chang

**Affiliations:** ^1^Department of Population Medicine and Diagnostic Sciences, Cornell University, Ithaca, NY 14580, USA; ^2^Division of Gastroenterology and Liver Disease, Case Western Reserve University, Cleveland, OH 44106, USA; ^3^Department of Clinical Studies, University of Guelph, Guelph, ON, Canada N1G 2W1

## Abstract

Aberrant false-positive reactions in negative-controls during ELISA testing for *Clostridium difficile* indicated the potential for false-diagnoses. Experiments with 96-well products showed a maximum peak of false-positive immunoassay reactions with the provided negative-control reagents after 5 refrigeration-to-room temperature cycles (*P* < 0.001), decreasing thereafter with additional refrigeration cycles. Because repetitive refrigeration causes a peak of false-positives, the use of single negative-controls per ELISA run might be insufficient to monitor aberrant preanalytical false-positives if immunoassays are subject to repetitive refrigeration.

## 1. Introduction

The diagnostic performance of ELISA immunoassays for the detection of toxins A and B of* Clostridium difficile* is still hampered by false-positive diagnoses [1–3]. Despite their recognized occurrence and the potential role of stool desorbing activity [4] and other host-associated factors [5], the mechanisms underlying false-positivity dependent on the immunoassays remain unclear. To prevent epidemiological bias, negative-controls must be used [6] to warn when false-positives occur due to product failure.

As per most manufacturers, every commercial ELISA needs two controls, one positive and one negative. Laboratories often run samples in small batches especially when diagnostic demands are low. Under those circumstances, ELISAs in 96-well formats may be subject to multiple refrigeration-to-room-temperature cycles (refrigeration cycles). Although manufacturers state that their 96-well products are stable if kept at constant refrigeration, this may not always be possible unless reagents are initially aliquoted and thereafter used one-aliquot-at-a-time. To date, commercial products often do not provide information regarding how many refrigeration cycles are tolerable and their effect on test performance.

Recently, we observed aberrant false-positives reactions in negative-controls, in a laboratory with long-standing research and diagnostic capabilities for* C. difficile* [7–10]. The false-positives occurred progressively as a 96-well format commercial immunoassay kit for the detection of* C. difficile* toxins A and B [11] was used to test small batches of fecal samples over time. Product handling was in accordance with product specifications [11]. Upon quality control examination of the product, 5 months prior to its expiration date, analysis indicated that the repetitive refrigeration of the ELISA wells could have been the cause of impaired performance. To date there are no available studies assessing the impact of repetitive refrigeration on false-positive performance in the negative commercial controls that are part of an ELISA kit. Here, we quantified the effect of repetitive refrigeration on false-positivity of negative-controls using a 96-well ELISA product that instructs to bring to room temperature all refrigerated reagents (liquid and dry ELISA wells) every time the product is needed for testing [12, 13].

## 2. Materials and Methods

To precisely quantify the hypothetical deleterious effect of refrigeration on the performance of negative-control reactions, a blinded two-factor experiment (number of refrigeration cycles, expiration status) was conducted with an ELISA product that instructed that all reagents should reach room temperature prior testing (Meridian Bioscience, Inc., Cincinnati, OH) [12, 13]. One ELISA kit purchased from an authorized vendor, kept under stable refrigeration, and valid for testing with 6 months prior to the expiration date was used for testing. Two other Meridian kits with 3 and 15 months after their expiry dates were available to verify if false-positives would occur after the product's expiry date. For each of the three kits tested, all available wells were randomly assigned to four groups (*n* = 4–11 wells/group), placed inside a 50 mL plastic tube with fifteen 0.5 cm perforations, and covered with aluminum foil to allow acclimation and protect from light. Each of the tubes was exposed to one of four conditions: 20, 10, 5, or 0 refrigeration cycles. Each cycle comprised alternate exposure to refrigeration (2 ± 5 h; 4°C) and room temperature (1 ± 0.2 h, 23°C, relative humidity of 70%). After 20 cycles, all wells (*n* = 105) were simultaneously tested using the corresponding liquid reagents, which were not subject to refrigeration changes. The negative-control reactions were performed using the provided negative-control commercial reagents, which are free of* C. difficile* toxins, following the manufacturer's instructions [12, 13]. To ensure homogeneous washing, prevent well-to-well spillage, and allow the systematic distribution of technical variability, random wells from all four groups were orderly alternated, leaving empty spaces in between, when placed on the 96-well washing tray. OD_450_ readings were used following the manufacturer's cutoff criteria to categorize reactions as positive or negative [12, 13].

## 3. Results

**Figure 1:**
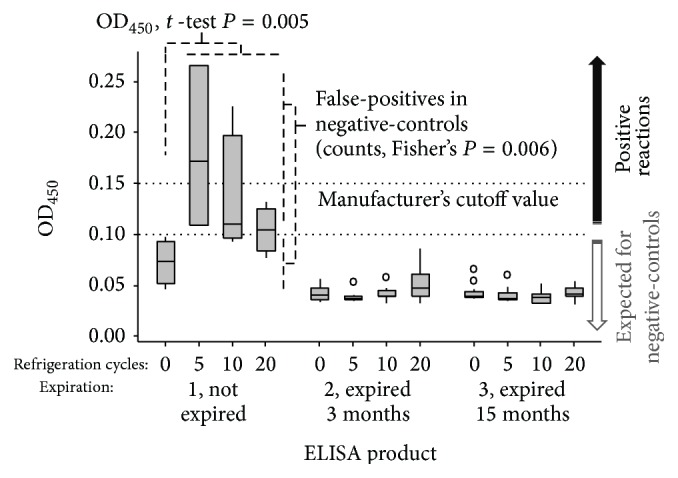
Refrigeration cycles increased the OD_450_ and false-positivity (convex correlation) in the ELISA wells of a valid nonexpired ELISA kit. The product insert of this ELISA kit instructed to bring all reagents to room temperature each time before testing. Positive-controls, not shown, performed as expected. The expired products yielded no positive or negative reactions as expected.

Analysis of OD_450_ data indicated that repetitive refrigeration had significant diagnostic deleterious effect on the performance in the negative-control reactions. Of primary concern, supporting the observation of aberrant positivity, there was a peak of false-positivity by the fifth refrigeration cycle (*P* < 0.001), which gradually decreased over 15 additional cycles (Figure 1). Positive-controls performed as expected (OD_450_ > 0.9; above 0.5 cutoff). As expected, none of the expired reagents provided any measurable reactivity indicating that false-positives are not random and that they do not occur in expired products.

## 4. Discussion

Appropriate negative-controls are fundamental in research and clinical practice [6], especially in small laboratory settings [2]. The present study quantified for the first time that repetitive refrigeration causes a peak of false-positives in the commercial ELISA negative-control reactions that should be negative because the reagents are free of* C. difficile* toxins. The cause of false-positivity is unclear but may be due to gradual deterioration of antigen binding affinity of antibodies coating the ELISA wells.

Our report highlights the importance of monitoring the appropriate performance of negative-controls to improve diagnostic accuracy, a concept that may apply to various immunoassays [3, 14]. The way the manufacturer's instructions are interpreted by laboratory technician(s) is critical and depends on the wording and quality of the recommendations provided. At the time of testing, the Meridian ELISA product insert specifically indicated to bring to room temperature all refrigerated reagents each time prior to testing [13]. Examining the word count and content for a comparable ELISA product [11, 12] indicated that instructions on “storage/handling of the ELISA product” reagents compared to the instructions on “storage/handling of the fecal specimens” for testing are arguably variable and nonspecific across immunoassays [11, 13]. Including updated technical information regarding product tolerance to repetitive refrigeration cycles and providing technical alternatives to laboratory users to minimize immunoassay exposure to unnecessary refrigeration cycles of the products would be advisable. Users would also benefit by aliquoting reagents for storage after unpacking a new product and ensuring that every ELISA test is performed with wells that have been refrigerated only once prior to use. The reduction of false-positive reactions during ELISA testing of fecal specimens, by preventing unnecessary refrigeration cycles and product deterioration, has to be accompanied by proper storage and handling of the fecal specimens [9, 15]

## 5. Conclusions

Although the findings of false-positivity induced by repetitive refrigeration apply to the immunoassay tested, it is possible as a proof of principle to infer that the use of one negative-control for each ELISA run might be insufficient to monitor aberrant preanalytical false-positives if this or other commercial immunoassays are subject to repetitive refrigeration.
